# Effect of sweet and caloric drinks on cardiac reactivity to slow-paced breathing in healthy adults

**DOI:** 10.1038/s41598-025-00980-w

**Published:** 2025-05-19

**Authors:** Maria Meier, Stephanie J. Dimitroff, Bernadette F. Denk, Eva Unternaehrer, Jens C. Pruessner

**Affiliations:** 1 University of Konstanz, Constance, Germany; 2 Child- and Adolescent Psychiatric Research Department, University Psychiatric Clinics Basel (UPK), University of Basel, Basel, Switzerland; 3 University of Montana, Missoula, MT USA; 4 Centre for the Advanced Study of Collective Behavior, Constance, Germany

**Keywords:** Blood glucose, Slow-paced breathing, Vagal activity, Heart rate variability, Pre-ejection period, Psychology, Medical research, Biomarkers

## Abstract

Cardiac reactivity is proposed to be a central indicator of autonomic functioning. While hyperglycemia plays a limited role in cardiac stress reactivity, it is unclear whether it may modulate cardiac reactivity in non-stressful situations. We investigated the effect of glucose on cardiac reactivity to a relaxing exercise, namely, slow-paced breathing (SPB). A total of 115 adults (age *mean* = 23.28 years, *SD* = 6.88; 76% female) either consumed a *sweet & caloric*, a *sweet*, a *caloric* drink*,* or pure *water* after baseline. Later, they performed a sustained attention test and SPB. Electrocardiography and impedance cardiography was obtained, and blood glucose and subjective relaxation were measured repeatedly. We analyzed changes in parasympathetic (root mean square of successive differences [RMSSD]) and sympathetic (pre-ejection period [PEP]) cardiac activity and subjective relaxation using growth curve models and performed correlational analyses. Hyperglycemia triggered cardiac PNS withdrawal and SNS activation. Despite this, SPB increased cardiac PNS activity and subjective relaxation and decreased cardiac SNS activity in all groups. Our results align with the autonomic space model and highlight the tight link between autonomic regulation and blood glucose homeostasis. Hyperglycemia might play a limited modulating role in cardiac reactivity to slow-paced breathing.

**Supplementary Information:**

The online version contains supplementary material available at 10.1038/s41598-025-00980-w.

## Introduction

Dysregulations of the autonomic nervous system have repeatedly been linked to deleterious health outcomes including chronic somatic diseases and psychopathology^1,2^. Cardiac markers like pre-ejection period (PEP)^3^ and vagally mediated heart rate variability (HRV)^4^ capture the activity of the two main branches of the autonomic nervous system—the sympathetic nervous system (SNS) and the parasympathetic nervous system (PNS) —at the level of the heart.

### Cardiac reactivity as indicator of autonomic functioning

Next to resting activity, cardiac reactivity is discussed as a central indicator of autonomic functioning. For example, cardiac PNS reactivity was proposed to reflect the capacity of an individual to adequately react to the environment^5,6^. Cardiac reactivity is typically operationalized as the change from baseline in response to a given demand. Thereby, reactivity can describe both, a relative decrease and a relative increase of activity. For example, cardiac stress reactivity—which typically goes hand in hand with an increase in subjective arousal^7^—is characterized by an increase in SNS and a decrease in PNS activity. In contrast, slow-paced breathing (SPB) —an exercise with marginally positive effects on mood^8^—has been shown to increase cardiac PNS activity^9–11^ but has little or a slightly decreasing effect on SNS activity^12^. To date, the exact meaning of cardiac reactivity is still under debate and more basic research is needed to elucidate determinants and modulators thereof^13^. One modulating factor may be glucose metabolism, which is tightly linked to autonomic control^14,15^.

### Effects of glucose on cardiac activity

Both the SNS and PNS are centrally involved in the regulation of glucose metabolism, ensuring that blood glucose levels are maintained within a narrow range^16,17^. While the PNS triggers insulin release^16^, the hormonal end products of the SNS—norepinephrine and epinephrine—counteract insulin action and mobilize energy^18^. Accordingly, a rise in blood glucose triggers cardiac PNS withdrawal^19,20^ and SNS activation^15,21,22^. While the effect of hyperglycemia on cardiac activity at rest is well established^16,17^, the impact of glucose on cardiac reactivity to tasks that are independent of the glucose challenge itself is only partly understood.

### Effects of glucose on task-dependent cardiac reactivity

Some studies investigated the effect of hyperglycemia on cardiac reactivity to stress. While glucose increased the endocrine response to psychosocial stress^23–28^, no significant effects on autonomic outcomes (i.e., heart rate or salivary alpha-amylase) were reported^25,27^. Furthermore, neither glucose nor sweetener modulated autonomic reactivity to challenging tasks^22^, and carbohydrate-rich meals were not found to modulate cardiac reactivity to the cold pressor test^29,30^. Of note, some of these studies utilized autonomic measures that are influenced by both branches of the autonomic nervous system (i.e., heart rate, salivary alpha-amylase). This critically limits the informative value regarding potential differential effects of glucose on cardiac SNS and PNS reactivity. Nevertheless, these results suggest that glucose might not modulate cardiac stress reactivity. However, the autonomic nervous system and the endocrine stress axis interact tightly^31^, and as such, it cannot be readily assumed that the results of glucose on cardiac stress reactivity generalize to non-stressful contexts. It is therefore less clear whether hyperglycemia might also modulate cardiac reactivity in non-stressful situations.

### The current study

The aim of the current study was therefore to investigate the effect of glucose on PNS and SNS reactivity to SPB. To do so, healthy adults were invited in a fasted state to control blood glucose levels. As the sweet flavor of glucose has the potential to modulate PNS reactivity through the activation of sweet taste receptors^32^, which play a crucial role in the sensation of nutrients and glucose homeostasis^33^, participants were randomly assigned to consume one of four different drinks that varied in sweetness and caloric content (*sweet & caloric*, only *sweet*, only *caloric*, or *water*). Shortly after, participants completed a sustained attention task^34^ to avoid ceiling effects of subjective relaxation and performed SPB (4 s inhale, 6 s exhale). Thereby, we employed a multisystemic approach by measuring cardiac SNS and PNS activity separately^13^. We used the root mean square of successive differences (RMSSD) to capture cardiac PNS activity^4,35^, and pre-ejection period (PEP) to capture cardiac SNS activity^3^.

### Hypotheses and analysis plan

Based on results implicating the impairing effects of hypoglycemia on PNS regulation^36^, we hypothesized that increasing blood glucose levels through drinks containing glucose would increase PNS reactivity to SPB. As SPB was not consistently found to trigger changes in PEP^12^, no specific hypotheses were formulated, but analogous analyses were run. To study the potential effects of glucose mood^37^, we further tested whether the drinks affected subjective relaxation. Lastly, we performed correlational analyses between RMSSD, PEP and blood glucose concentrations.

## Results

### Preliminary analyses and manipulation checks

Descriptive statistics and sample characteristics are summarized in Table 1. The groups *sweet & caloric,* and *water* were exposed to a higher room temperature and higher humidity as compared to the groups *sweet,* and *caloric*. Further, participants in the groups *sweet & caloric,* and *water* had a lower blood glucose baseline. RMSSD baseline was significantly related to humidity, *r*(113) = − 0.27, *p* = 0.004, and temperature, *r*(113) =  − 0.22, *p* = 0.018, but not blood glucose baseline, *r*(113) = 0.08, *p* = 0.369; PEP baseline was significantly associated with humidity, *r*(104) = 0.21, *p* = 0.030, but neither with temperature, *r*(104) = 0.03, *p* = 0.723, nor with blood glucose baseline, *r*(104) = -0.13, *p* = 0.172. For this reason, we controlled for temperature and humidity in the analyses focusing on RMSSD, and for humidity in the analyses focusing on PEP.

**Table 1:** Descriptive statistics of the experimental groups.

variable	Sweet & caloric (*n* = 33)	Sweet (*n* = 26)	Caloric (*n* = 25)	Water (*n* = 31)	*p*-value
age (years)	23.88 (6.87)	22.96 (7.96)	21.92 (3.53)	24.00 (7.87)	0.665
sex: male/female (in %)^a,c^	27.3 / 72.7	23.1 / 76.9	24.0 / 76.0	22.6 / 77.4	0.973
hormonal status of women: follicular/luteal/OC (in %)	39.1/34.8/26.1^d^	33.3/16.6/50.0^e^	47.3/21.1/31.6	30.4/43.5/26.1^d^	0.680
BMI (kg/m^2^)	21.96 (2.48)	21.73 (2.21)	22.03 (2.71)	22.74 (2.42)	0.425
Blood glucose baseline (mg/dl)	95.21 (9.93)	107.62 (16.13)	103.24 (10.74)	95.16 (11.40)	** < 0.001**
RMSSD baseline	35.39 (16.49)	47.18 (33.71)	51.80 (31.05)	45.68 (22.33)	0.103
PEP baseline	109.92 (9.49)	104.55 (10.36)	103.03 (20.57)	109.51 (12.73)	0.169
Respiration rate baseline (cpm)	15.23 (2.90)	15.29 (2.98)	15.03 (3.16)	15.71 (2.97)	0.854
Session start: 3/5 p.m.^a^	19/14	11/15	10/15	17/14	0.448
Room temperature (°C)	23.37 (2.29)	20.82 (2.43)	21.17 (1.95)	22.74 (1.44)	** < 0.001**
Room humidity (%)	40.06 (10.20)	33.73 (16.03)	23.64 (5.23)	40.23 (9.34)	** < 0.001**

If not otherwise specified, a one-way Analysis of Variance by *drink* was calculated to test whether the four groups differed with respect to the listed variables. In these cases, data is expressed as *mean* ± *SD*. OC = oral contraceptive use. BMI = Body Mass Index.

^a^Pearson’s Chi-squared test was calculated to test whether *conditions* differed with respect to the listed variable. ^b^variable “sex” was assessed in self-report as assigned sex at birth. ^c^variable estimated based on self-reported average cycle duration, date of last cycle start, and an estimated luteal phase duration of 14 days. ^d^1 missing. ^e^2 missing.

Significant values are in (bold).

The groups did not differ in hunger, *F*(3, 110) = 2.32, *p* = 0.079, *partial eta squared* = 0.06, or thirst, *F*(3, 110) = 0.15, *p* = 0.927, *partial eta squared* < 0.01, when entering the experiment. The groups differed significantly in how much they liked the consumed drink, *F*(3, 109) = 13.45, *p* < 0.001, *partial eta squared* = 0.27. The *water* group liked the drink significantly more (mean = 66.00, *SD* = 26.60) as compared with all other groups (*sweet:* mean = 34.20, *SD* = 27.30; *caloric*: mean = 41.30, *SD* = 24.50, *sweet & caloric*: mean = 26.00, *SD* = 26.20). Further, the groups differed significantly in how sweet they perceived the consumed drink, *F*(3, 109) = 325.56, *p* < 0.001, *partial eta squared* = 0.90. *Water* was rated as the least sweet (mean = 4.31, *SD* = 5.59) and both drinks containing sweetener as the most sweet (*sweet:* mean = 87.10, *SD* = 11.70, *sweet & caloric:* mean = 90.90, *SD* = 8.91). The *caloric* drink was perceived to be sweeter than *water* (mean = 58.20, *SD* = 19.20), but not as sweet as the drinks containing sweetener.

**Fig. 1:**
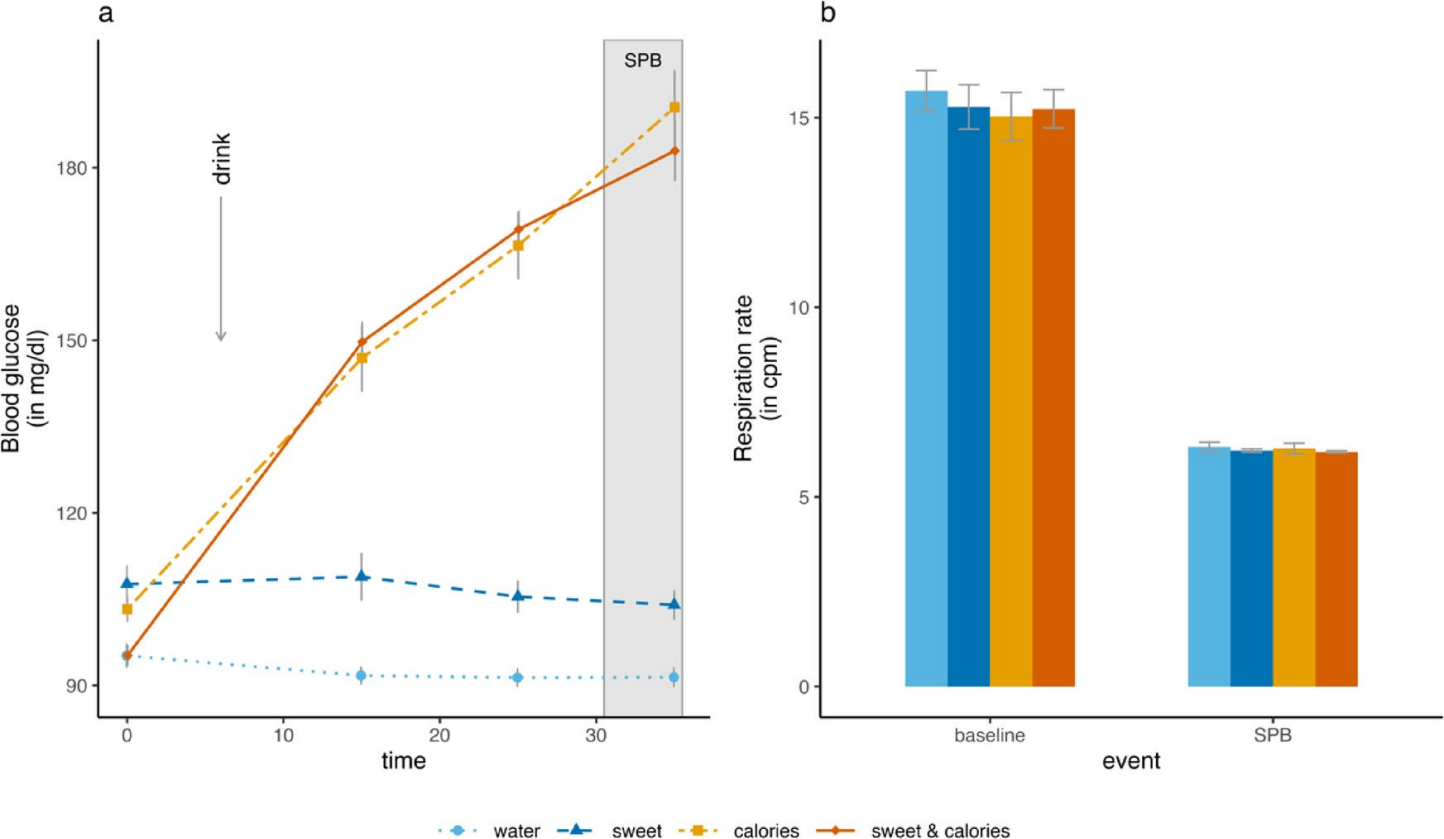
Changes in (**A**) blood glucose concentrations over time, and (**B**) respiration rate. *SPB* slow-paced breathing.

As expected, blood glucose levels increased in response to caloric drinks, but remained low in the groups consuming the *sweet* drink, or pure *water.* In the growth curve model predicting blood glucose concentrations (460 observations nested in 115 participants), the inclusion of random intercepts, random slopes, a linear and quadratic trend of *time*, the main effect of *drink*, and the *drink* by *time* interactions led to significant increases in model fit (cf. supplementary material Table S1). The final model (marginal *R*^2^ = 0.84, conditional *R*^2^ = 0.92) showed that both caloric drinks significantly increased blood glucose concentrations compared with *water* (*sweet & caloric* x *time*: *b* = 728.33, *SE* = 45.81, *p* < 0.001, *caloric* x *time*, *b* = 713.84, *SE* = 49.23, *p* < 0.001, cf. supplementary material Table S2). Changes in blood glucose concentrations over time are depicted in Fig. 1A.

Respiration rate was significantly lower during SPB (*mean* = 6.25, *SD* = 0.47) as compared with the baseline (*mean* = 15.33, *SD* = 2.97), *t*(110) = 30.44, *p* < 0.001, *d* = 2.89, and normalized directly after the exercise (*mean* = 14.52, *SD* = 2.49). Respiration rate during SPB was significantly higher than the predefined respiration rate of 6 breaths per minute, *t*(110) = 5.53, *p* < 0.001, *d* = 0.52, but did not differ between *drink* groups, *F*(3, 107) = 0.41, *p* = 0.749, *partial eta squared* = 0.01, see Fig. 1B.

### Effect of drinks on RMSSD

RMSSD tended to decrease stronger after caloric drinks compared with water drinks, but these differences vanished during SPB, which induced an increase in RMSSD in all groups. The RMSSD response to SPB was not modulated by the drinks and RMSSD did not significantly differ between *drink* groups during the exercise, *F*(3, 101) = 1.33, *p* = 0.268, *partial eta squared* = 0.04.

The growth curve model predicting RMSSD (575 observations nested in 115 participants) was controlled for humidity and temperature. The inclusion of a random intercept, a linear, quadratic and cubic trend of *time*, the autoregressive covariance structure (AR1), and the main effect of *drink* increased the model fit significantly (cf. supplementary material Table S3).

The final model (conditional *R*^2^ = 0.75, marginal *R*^2^ = 0. 09) indicated a significant quadratic trend of *time, b* = − 84.27, *SE* = 27.73, *p* = 0.003. RMSSD was significantly higher during SPB (*mean* = 65.00, *SD* = 22.37) as compared with the baseline (*mean* = 44.40, *SD* = 26.41), *t*(107) =  − 11.42, *p* < 0.001, *d* = − 1.11. After the exercise, RMSSD returned to baseline levels (*mean* = 48.87, *SD* = 25.91).

Further, the growth curve model indicated a significant main effect of *sweet & caloric*, *b* = − 14.99, *SE* = 5.23, *p* = 0.005, and a significant *time*^*3*^ x *sweet & caloric* interaction effect, *b* = − 98.44, *SE* = 41.68, *p* = 0.019. The group s*weet & calories* displayed stronger RMSSD dynamics as compared with the *water* group. Compared to *water* (mean = 63.94, *SD* = 34.53)*,* s*weet & caloric* (mean = 41.48, *SD* = 18.11) displayed a significantly lower RMSSD directly after drink consumption, *t*(44.70) = 3.23, *p* = 0.002, *d* = 0.82. Furthermore, *sweet & caloric* (mean = 32.84, *SD* = 17.19) had a lower RMSSD during the d2 test than *water* (mean = 51.99, *SD* = 26.21), *t*(51.28) = 3.43, *p* = 0.001, *d* = 0.87. A similar trend for stronger RMSSD dynamics following drink consumption was observed in the *caloric* group (non-significant *time*^*2*^ x *caloric* interaction: *b* = 68.93, *SE* = 41.51, *p* = 0.097).

**Fig. 2:**
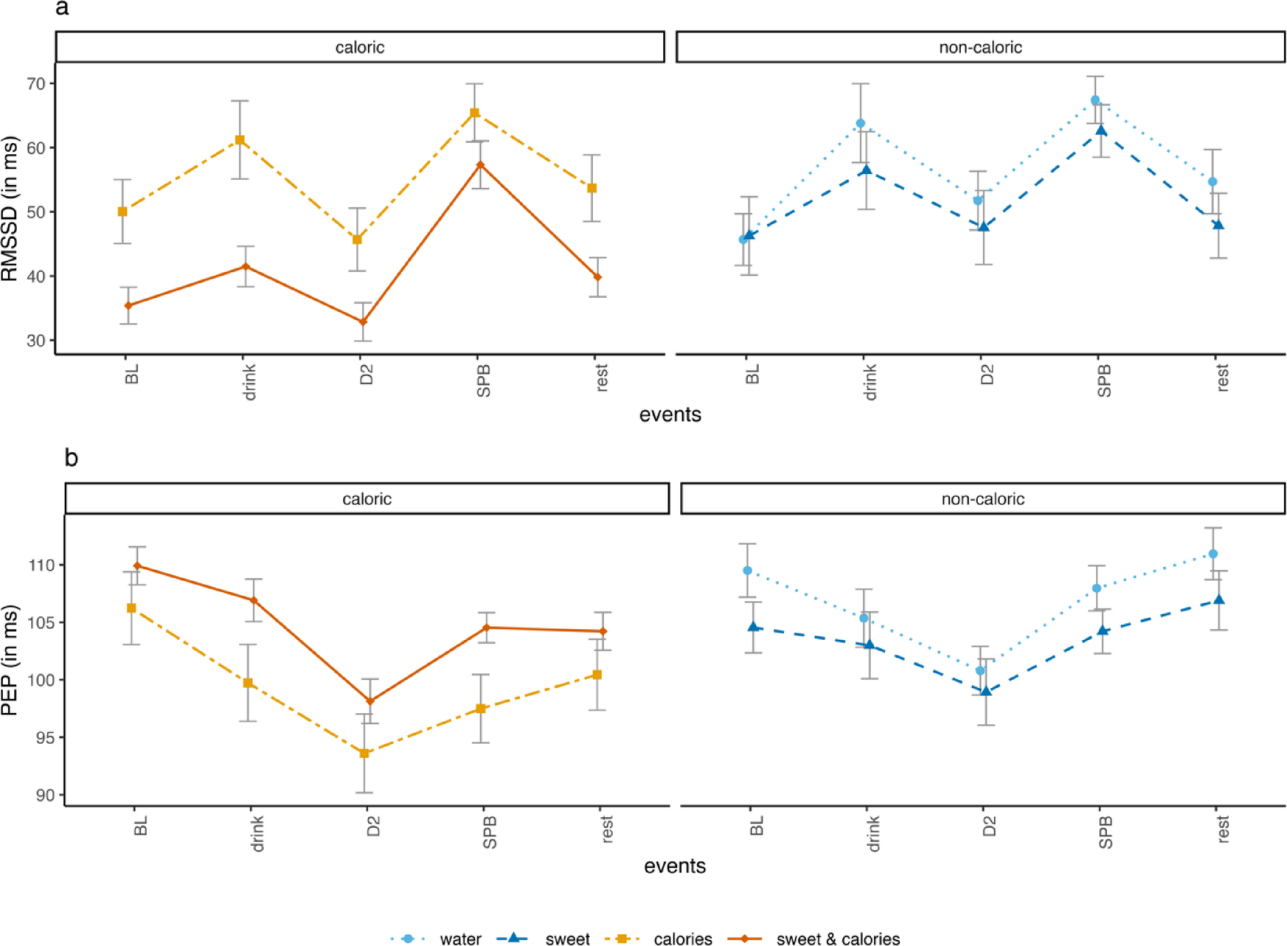
Changes in (**A**) root mean square of successive differences (RMSSD) as an indicator of parasympathetic activity, and (**B**) pre-ejection period (PEP) as an indicator of sympathetic activity in the different drink groups.

The detailed results can be retrieved from the supplementary material (Table S4) and changes in RMSSD over time are depicted in Fig. 2A.

### Effect of drinks on PEP

SPB decreased PEP in all groups and both caloric drinks induced a linear decrease of PEP over time. Model fit of the growth curve model predicting PEP (535 HR observations nested in 107 participants), in which we controlled for humidity, increased significantly when including random intercepts, a linear and quadratic trend of *time,* the covariate, and the *condition* by *time* interactions (cf. supplementary material Table S5).

The evaluation of the final model (marginal *R*^2^ = 0.33) showed a significant quadratic trend of *time, b* = 70.67, *SE* = 11.77, *p* < 0.001. Across groups, PEP was significantly higher during baseline (*mean* = 107.32, *SD* = 13.53) as compared with SPB (*mean* = 103.42, *SD* = 12.28), *t*(103) = 4.48, *p* < 0.001, *d* = 0.44, and increased after cessation of the exercise (*mean* = 105.27, *SD* = 13.98). Both caloric drinks led to a significant linear decrease in PEP over time (*time* x *caloric*, *b* = *-*62.80, *SE* = 18.76, *p* < 0.001, and *time* x *sweet & caloric*, *b* = − 62.57, *SE* = 16.86, *p* < 0.001; cf. supplementary material Table S6). Changes in PEP over time are depicted in Fig. 2B.

### Effect of drinks on subjective relaxation

Subjective relaxation decreased in response to the d2 and increased in response to the SPB; the drinks did not significantly modulate this response. The model fit of the growth curve predicting subjective relaxation (690 observations nested in 115 participants) increased significantly upon inclusion of random intercepts and linear, quadratic, and cubic trends of *time* (cf. supplementary material Table S7). The time trend in the final model (marginal *R*^2^ = 0.16) was best described by a cubic trend, *b* = 110.33, *SE* = 28.39, *p* < 0.001 (cf. supplementary Fig. S1). We neither observed a significant effect of *drink*, nor a *drink* by *time* interaction (cf. supplementary material Table S8 for detailed list of model coefficients).

Compared to the rating assessed prior to baseline (*mean* = 38.20, *SD* = 14.80), subjective relaxation decreased in response to the d2 test (*mean* = 21.19, *SD* = 13.90), and increased in response to SPB (*mean* = 45.99, *SD* = 17.83). Ratings after SPB were significantly higher as compared with the baseline, *t*(114) =  − 4.00, *p* < 0.001, *d* = − 0.37, but did not differ between *drink* groups, *F*(3, 111) = 1.35, *p* = 0.263, *partial eta squared* = 0.04.

### Correlation between RMSSD, PEP and blood glucose concentrations

Higher blood glucose concentrations before the d2 test were related to lower RMSSD during the d2 test, *r*(113) =  − 0.21, *p* = 0.022. Further, higher blood glucose concentrations prior to SPB were related to lower PEP during SPB, *r*(104) =  − 0.22, *p* = 0.024.

## Discussion

In this experiment, we examined the effect of glucose on cardiac PNS and SNS reactivity in healthy adults. To differentiate the effects of sweet taste from the caloric load, participants received a either a *sweet & caloric,* a *sweet,* a *caloric* drink*,* or pure *water* before performing a sustained attention test and SPB*. Caloric* drinks triggered a counterregulatory cardiac response. The sustained attention test transiently decreased subjective relaxation as well as cardiac PNS activity and increased cardiac SNS activity, which may related to norepinephrine release^38^. Neither cardiac reactivity nor changes in subjective relaxation to SPB were modulated by hyperglycemia. While previous research consistently showed that autonomic outcomes were not modulated by increased glucose availability in challenging or stressful settings^23,25,27,30^, our results suggest that these findings may be generalizable to non-stressful contexts like SPB.

Caloric drinks increased blood glucose concentrations, which remained hyperglycemic throughout the experiment. They led to cardiac PNS withdrawal^19,20^, and an increase in cardiac SNS activity^15,21,22^, which may have been mediated by hypothalamic pathways^39^ and related to a rapid insulin peak approximately 5–10 min after glucose consumption^40,41^. While some studies reported increased resting PNS activity directly after intravenous glucose or insulin injection^42^, our results are in line with the well described counterregulatory response to hyperglycemia in healthy adults^16,17^.

On average, SBP successfully lowered participants’ respiration rate, even though it was slightly higher than 6 cycles per minute. Personalizing the rate based on individual’s respiration baseline could have eased the execution for participants^43^, but would have required a more advanced technical setup. In line with previous results^9,11,44,45^, SPB increased subjective relaxation, but the effects were rather modest.

Despite the counterregulatory response to hyperglycemia, SPB increased cardiac PNS activity and subjective relaxation, and decreased cardiac SNS activity across all groups. While some found that SPB had a negligible effect on cardiac SNS activity^12^, our results suggested otherwise. Despite this, our results replicate previous effects of SPB that were observed without a deliberate manipulation of blood glucose levels^8^. They extend these findings by showing that hyperglycemia in healthy adults may only play a limited modulatory role in this context, a finding that generalizes across stressful and non-stressful scenarios.

In rodents, increased glucose availability has been shown to facilitate acetylcholine synthesis and release^46^, suggesting a tight link between hyperglycemia and the cholinergic pathway. In healthy adults, lower nocturnal cardiac PNS activity has been related to higher fasting blood glucose and glycosylated hemoglobin (HbA1c)^47^—a finding matching the reported impaired PNS modulation in (pre)diabetic populations^48,49^. Despite the well-established links between glycemic indices and cardiac activity at rest, effects of glucose on cardiac reactivity are investigated less frequently, even though reactivity markers may convey important information about cardiac functioning: They have been proposed to reflect the functionality of the system to adapt to the environment^5^—a feature inherently important for higher-order processes like emotion regulation and social functioning. In light of this, further studies employing a multisystem approach, and investigating determinants and modulators of cardiac reactivity are highly warranted^50^. As the autonomic changes observed following SPB are mainly driven by the (physiological) coupling of respiration with the heartbeat^8^, it may be worthwhile to explore the effects of increased glucose availability on cardiac reactivity in other, more socially driven scenarios in the future. Emotion regulation is strongly linked to cardiac PNS activation^51^, and seems to be associated with glucose availability^52^. It may thus be speculated whether hyperglycemia might be prone to impact the cardiac response emotional or social stimuli.

The drinks did not modulate changes in subjective relaxation. This is in line with previous findings showing no changes in subjective relaxation after sweet or caloric drink administration^53^, and no modulation of subjective stress ratings after glucose consumption in threatening situations^26,27^. While sugar-sweetened tea had a calming effect on individuals undergoing acute stress^37^, further research is needed to determine why and under which circumstances sweets may modulate affect and act as comfort food in stressful situations^54^.

The current results must be interpreted considering some limitations. First, caloric and/or sweet drinks were perceived as being sweeter than *water*. While the *caloric* drink was perceived as moderately sweet, drinks containing artificial sweetener were rated as very sweet. Consequently, the *caloric* drink failed to present an adequate “caloric but non-sweet” control. While the congruency between sweetness and caloric content plays a role in reward processing^55^, the match between sweet taste and caloric load did not seem to be relevant in the current experiment. Nevertheless, it may be worthwhile to consider both dimensions as potential mediators of psychophysiological responses in future studies. For this, we advise against maltodextrin as a “tasteless” control. Second, due to contact restrictions, participants were asked to set event markers at prescheduled timepoints, but some failed at doing so which is why some recordings could not be analyzed meaningfully. This caused unusual dropouts despite good data quality. Also, the ICG signal was noisy in a substantial number of recordings, which led to further exclusions. The lower sample size limited statistical power. Third, our sample consisted predominantly of healthy, young females. As sex and age have been reported to modulate PNS inhibition to carbohydrates^56^, follow-up studies including more diverse or sex-balanced samples are warranted to test the generalizability of these results. Fourth, participants completed an online screening in which they self-reported important health-related outcomes like chronic illness and medication use. However, this might introduce inaccuracies if participants are unaware of certain conditions or choose to conceal information relevant for the correct interpretation of the physiological outcomes. In our case, two participants showed very frequent irregular beats throughout the ECG recording which was independent of the experimental manipulations. As a result, we excluded them from our analyses. However, these cases signify that relying on self-reported “health” may be a shaky venture. Lastly, blood glucose baseline levels differed between groups, which might be related to the season in which participants were tested^57^. Also, room temperature and humidity differed between groups and were related to our cardiac outcomes, which is why we accounted for these differences statistically. We cannot rule out that the differences might have biased our results.

Despite these limitations, this study also has considerable strengths. We used a multisystem approach to study the effects of glucose on cardiac PNS and SNS reactivity in a well-controlled laboratory setting. Examining the (transient) effects of common macronutrients like glucose on cardiac reactivity in healthy populations can further our understanding of the (co)regulation of the metabolic and the autonomic nervous systems and inform about important covariates in settings with (ambulatory or laboratory) cardiac assessments^58^. While caloric drinks activated cardiac SNS and inhibited PNS activity, cardiac reactivity to SPB remained unaffected. These findings provide further evidence for the autonomic space model^59^, highlighting that cardiac PNS reactivity is not impeded by SNS activation, and cardiac activity of one branch of the autonomic nervous system cannot be inferred from the other.

## Method

### Participants

The project was approved by the Ethics Committee of the University of Konstanz and was carried out in accordance with the Declaration of Helsinki. Healthy adult participants were recruited via flyers placed at the facilities of the University of Konstanz, via the participant pool management software of the University of Konstanz (SONA Systems) and via social media. Participants completed an online eligibility screening before being invited to the laboratory to control the influence of variables known to impact cardiovascular regulation^35,60^.

Exclusion criteria were: (1) age < 18, (2) Body Mass Index (BMI) indicating underweight (< 18.5 kg/m^2^) or obesity (> 30 kg/m^2^), (3) lack of German language skills, (4) acute or persistent medication intake affecting the autonomic nervous system (e.g., psychopharmaceutic or anti-histaminic medication), (5) working nightshifts, (6) engaging in competitive sports due to effects of extensive physical exercise on cardiac functioning, (7) being on a diet or deliberately avoiding sugar in the diet, (8) regular smoking (> 5 cigarettes per day), (9) illegal drug consumption within last two weeks, or report excessive alcohol consumption (more than four drinks on more than one day per week), (10) being allergic or intolerant to sugar or sugar substitutes, (11) suffering from a chronic disease (e.g., cardiac disease, neurological disease, metabolic disease), (12) clinically relevant symptoms of depression (indicated by Beck’s Depression Inventory II sum score > 19)^61^. For women, menopause entry and pregnancy were additional exclusion criteria. Due to the ongoing COVID-19 pandemic, participants in a risk group had to be excluded to comply with the regulations of the University of Konstanz.

The study comprised the between-subject factor *drink* (sweet & caloric, sweet, caloric, water) and the within-subject factor *time*, as the physiological outcome measures (i.e., RMSSD, PEP, and blood glucose concentrations) were assessed continuously repeatedly. Prior to data collection, we estimated the sample size needed to achieve 95% statistical power using G*Power^62^. The analysis was based on the within-between interaction effect, in which we planned to compare RMSSD / PEP trajectories (five measures) of the four *drink* groups. We assumed a small effect (*f* = 0.15), a correlation among repeated measures of *r* = 0.5, and a non-sphericity correction factor of 1. Using these estimates, we planned to test a total sample of *N* = 120 (*n* = 30 in each group).

**Fig. 3:**
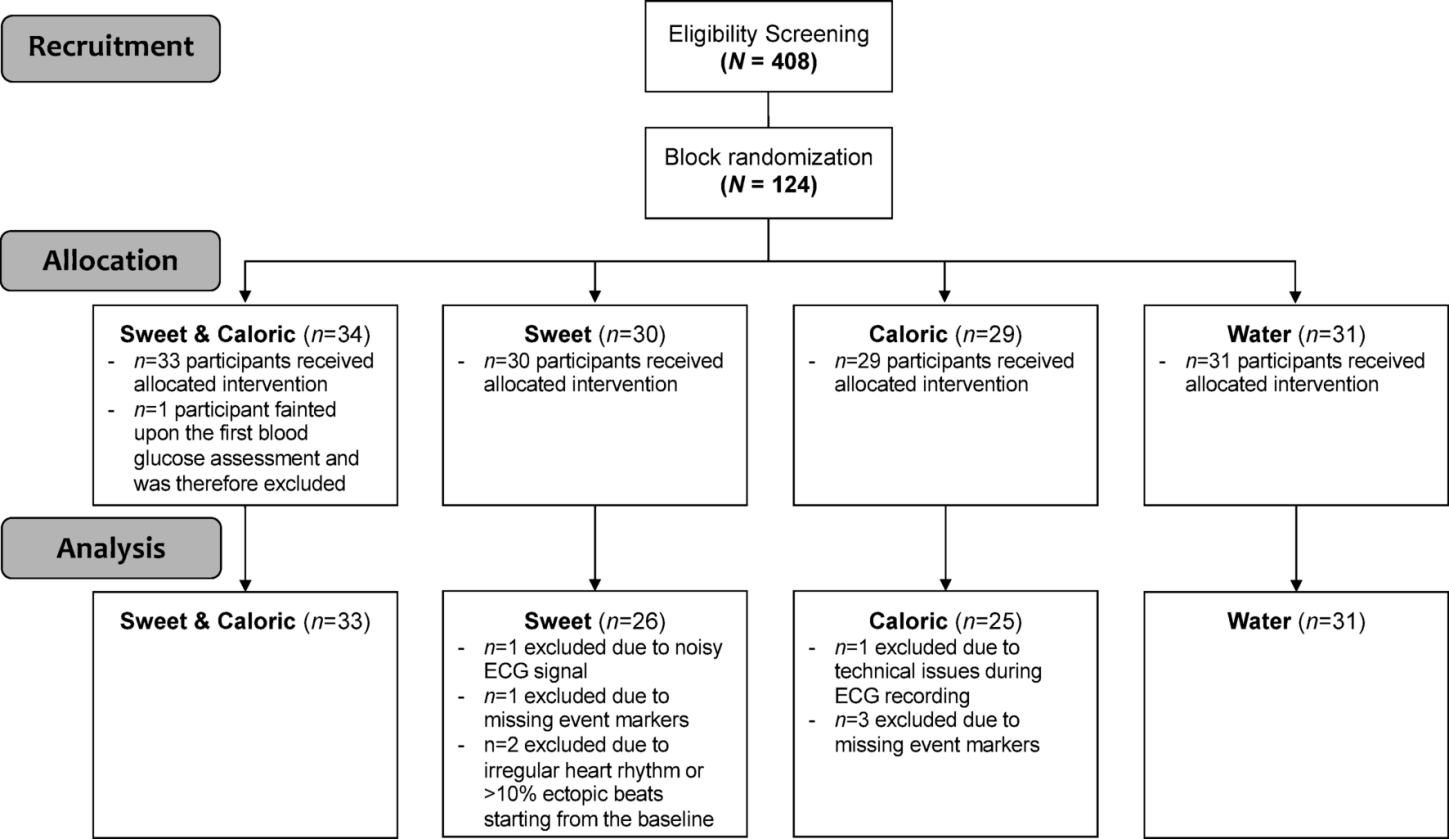
Flow diagram visualizing the sample of the study.

To account for potential dropouts, 124 eligible adults (age *mean* = 23.26 years, *SD* = 6.61 years; 73.39% female) participated. After the exclusion of *n* = 9 participants during the HRV preprocessing (for reasons see Fig. 3), we arrived at a final sample of *n* = 115 (age *mean* = 23.28 years, *SD* = 6.88 years; 75.65% female) that was used for the statistical analysis. The majority of participants identified themselves as European (88.7%).

A sample flow diagram is depicted in Fig. 3. For PEP analysis, *n* = 16 participants from the initial sample had to be excluded due to poor data quality, which is why these results are based on a sample of *n* = 107 (age *mean* = 23.28 years, *SD* = 6.84 years; 76.64% female).

### Experimental procedure

All sessions were performed at 3 or 5 p.m. to control for circadian influences^63,64^, and lasted 75 min. Participants were required to refrain from food and drinks (other than water) for 4 h before testing^65^. Further, they were asked to avoid physical exercise on the day of the experiment, not to smoke prior to the session, and to follow a normal sleep routine^35^.

To comply with the university’s contact regulations during the COVID-19 pandemic, the experimenter and the participant sat in two different rooms and communicated via video call. If the experimenter entered the testing room (e.g., for the blood glucose measurements), the experimenter and participant wore face masks. Before each session, the experimenter took note of the room temperature and relative humidity of the testing environment using a thermo-/hygrometer (Temeo Hygro indicator, Bresser, Rhede, Germany).

After welcoming participants, they were given the opportunity to use the bathroom^66^. Then, they gave written informed consent, attached seven electrodes of a portable electrocardiogram (ECG) and impedance cardiography (ICG) device to their skin (following the guidance of the experimenter who checked the quality of the signal prior to starting the recording), and were introduced to the Affect Grid^67^ that was used to measure changes in subjective relaxation during the experiment. Participants were instructed how to set event markers on the ECG/ICG device, and to sit as still as possible during the experiment^35^.

**Fig. 4:**
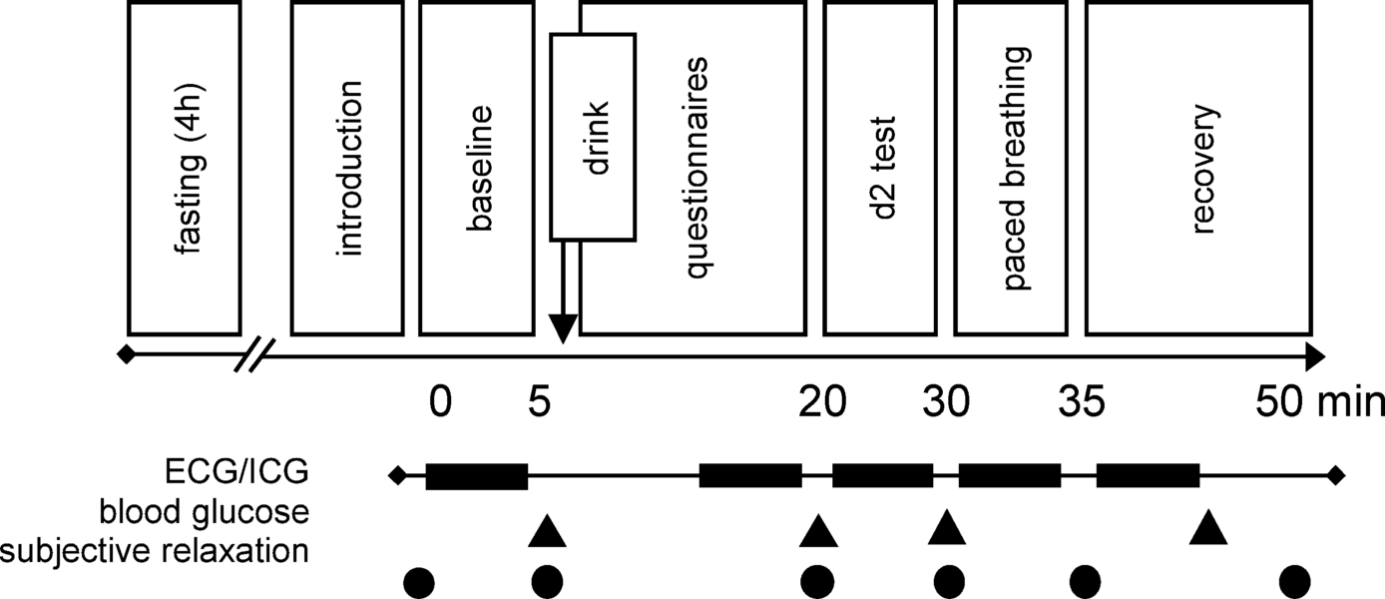
Experimental procedure. ECG/ICG = electrocardiogram/impedance cardiography.

The session started with a relaxation rating and the assessment of a physiological baseline as well as state measures of hunger and thirst. The baseline was recorded in silence in an upright sitting position with both feet placed on the ground (knees at 90° angle), the hands placed on the tights, and open eyes for 5 min. After a second relaxation rating and a first blood glucose measurement, participants consumed one of four different drinks and completed questionnaires. After 15 min, when glucose was absorbed into the bloodstream^68^, a third relaxation rating and a second blood glucose measurement were performed. To raise participants’ subjective arousal levels and avoid ceiling effects of subjective relaxation prior to SPB^69^, the d2 test (a cancellation test measuring sustained attention under time-pressure; Steinborn et al., 2018^34^) was performed. After a relaxation rating, blood glucose concentration was measured a third time. Then, SPB was introduced and carried out for 5 min. After another relaxation rating and a recovery period of 5 min, a last blood glucose measurement was conducted, and participants filled in questionnaires and rated their subjective relaxation level before being debriefed. Participants received 15€ or course credit for participation. The experimental procedure is depicted in Fig. 4.

### Tasks and measures

#### Drinks and experimental conditions

Participants were assigned to consume one of four different drinks that varied in *sweetness* (sweet, not sweet) and *energy content* (caloric, non-caloric).

The basis of all drinks was 300 ml of still mineral water. To manipulate the energy content, we added 75 g of maltodextrin with a dextrose equivalent of 19 and a high glycemic index (GI) of ~ 85. Maltodextrin is perceived as almost flavorless and absorbed rapidly into the bloodstream. To manipulate sweetness, we added 5.6 g of Natreen Classic liquid sweetener (Jacobs Douwe Egberts GmbH, Amsterdam, Netherlands). As a match between sweetness and energy content is particularly rewarding^55^, sweet taste matched the energy content of 75 g of sugar. By using two distinct substances to manipulate taste and caloric content, we aimed at maximizing the comparability of the different drinks in terms of taste and/or sugar absorption rate. All drinks were prepared by a third person and cooled at a temperature of approximately 8 °C.

The experimenter, and the participant were blind to the drink content prior to consumption. The assignment of participants to drinks was conducted within two blocks: *sweet & caloric* and *water* were assessed in the summer term, while *sweet* and *caloric* were assessed in the winter term of 2021. The assignment of participants within each block was random.

#### Slow-paced breathing

Participants performed a visually guided SPB exercise, in which inhalation and exhalation periods were paced to a fixed breathing ratio of 6 cycles per minute^9,11^. The task was guided by an in-house application displaying a blue circle that became bigger (inhale) for 4 s and smaller (exhale) for 6 s (Bae et al., 2021) in front of a white background (cf. video in the supplemental material) presented on an Apple MacBook Pro (15″). Before execution, participants watched a 2-min instructional video, during which the right sitting position (comfortable seat, both feet placed on the ground, hands placed on the upper tights), and the task were explained. Any remaining questions were resolved and the SPB was performed in silence for 5 min.

#### Physiological measures

*Blood glucose concentrations* Blood glucose concentrations (mg/dl) were measured from capillary blood of the fingertip at four timepoints using disposable lancets (Roche Diabetes Care, Mannheim, Germany) and a glucometer (A. Menarini Diagnostics, Berlin, Germany).

*Electrocardiogram and impedance cardiography.* An electrocardiogram (ECG) and an impedance cardiography (ICG) for the assessment of cardiac activity and respiration rate was obtained using seven spot electrodes (ECG electrodes ASF50, Asmuth Gmbh, Minden, Germany) and a portable MindWare Mobile device (Mindware Technologies, Gahanna, OH) with a sampling rate of 500 Hz. The electrode setup combined the standard Lead II with the standard tetrapolar system (Sherwood et al., 1990^3^). We defined five events of interest for the HRV and PEP analysis, each lasting 5 min (see black blocks in Fig. 4): (1) the baseline, (2) the questionnaire phase after drink consumption, (3) the d2 test, (4) the SPB exercise, (5) the recovery. Analogous to previous work (e.g., Stone et al., 2020^50^), we analyzed HRV and PEP in 1 min intervals (windowing) and averaged the intervals across each event. This approach has previously been shown to closely approximate averages of longer recordings^4^, while allowing to exclude noisy data minute-wise if needed.

*Heart rate variability* Analysis of the raw ECG signal was performed using MindWare Heart Rate Variability Analysis Software version 3.2.3. Data was manually inspected; artifacts were removed, and ectopic beats were corrected manually. For each event, we calculated mean RMSSD as an index of vagally mediated heart rate variability^35^. Missing RMSSD values were imputed using the mean of the respective event and outliers were winsorized prior to the statistical analysis.

*Pre-ejection period* Analysis of the impedance signal (Z0 and its first derivate dZ/dt) was performed using MindWare Impedance Cardiography Analysis Software version 3.2.13. Ensemble averages were calculated for 1-min epochs. PEP was calculated as the time between the Q wave of the ECG and the B point of the dZ/dt signal located using the Percent of dZ/dt Time + C method^70^. If needed, Z peak was corrected manually, and B was recalculated. X was placed at the minimum within a physiological plausible time window following R (Framingham method). For each event, we calculated mean PEP as an index of SNS activity. Prior to the statistical analysis, missing PEP values were imputed using the mean of the respective event and outliers were winsorized.

*Respiration rate* Respiration rate (in breaths per minute, bpm) was estimated using the impedance signal (Z0)^71^.

#### Self-report measures

Participants rated their mood (displeasure/pleasure, and sleepiness/arousal) six times (see black circles in Fig. 4) using the Affect Grid^67^. Values on each dimension ranged from 1 to 9, and higher values indicated higher arousal, or higher pleasure respectively. We multiplied the inverted arousal ratings with the pleasure ratings to obtain single item scores reflecting subjective relaxation^72^. Subjective relaxation ranged from 1 to 81, with higher scores indicating higher relaxation.

At the beginning of the experiment, participants indicated their current hunger and thirst on a visual analog scale that ranged from 0 (not at all) to 100 (very much). Using similar visual analog scales, participants were furthermore asked to rate how much they liked the drink and how sweet they perceived it. These ratings were used for descriptive purposes.

### Statistical analysis

Statistical analyses were conducted using R version 4.4.0 (R Core Team, 2024^73^) with RStudio version 2024.4.2.764^74^, and the packages *nlme*^75^, *dplyr*^76^, *reshape2*^77^, psych^78^, *sjPlot*^79^*, performance*^80^*,* and *apa*^81^. Graphs were created using *ggplot2*^82^ and *patchwork*^83^. The level of significance was set to *alpha* = 0.05.

We conducted multiple one-way Analyses of Variance (ANOVAs), and Chi-squared tests to test whether the *drink* groups differed in demographic variables, cardiac, respiratory, and blood glucose baseline levels or psychometric properties. Variables that differed significantly between groups and were significantly related to the outcome variable of interest^84^ were added as control variables to the confirmatory analyses. Using ANOVAs, we tested whether the drink groups differed in current hunger and thirst at the beginning of the experiment and whether the groups differed regarding drink liking and perceived sweetness. Using *t-*tests, we further tested whether SPB led to a significant reduction in respiration rate compared to baseline and whether participants were able to follow the predefined respiration rate of 6 breaths per minute.

Whenever we tested the effect of *drink* on repeated measure variables (i.e., blood glucose concentration, subjective relaxation, RMSSD, and PEP), we used a growth curve approach within a multilevel modeling framework and considered individual baseline differences (random intercepts) and differences in trajectories over time (random slopes) in our model^85^. In all models, we used a stepwise approach and included a linear, quadratic, and cubic fixed effect of time, and a first-order autoregressive covariance structure (AR1), if they significantly improved the model fit as indexed by Likelihood Ratio tests. Then, covariates, the main effect of *drink* (reference group: *water*), and the *drink* by *time* interaction effects were added. We evaluated the final model to obtain the coefficients of specific contrasts. We computed marginal *R*^2^ to quantify the variance explained by the fixed factors, and conditional *R*^2^ to quantify the variance explained by fixed and random factors. Finally, we computed Pearson’s correlations of RMSSD, PEP and blood glucose concentrations.

## Electronic supplementary material

Below is the link to the electronic supplementary material.

Supplementary Material 1

## Data Availability

Data of this project is openly available at https://osf.io/qdhjr/files/osfstorage. Raw IBI data can be requested from the first author for additional analyses. A preprint of this manuscript had been published on PsyArxiv: https://psyarxiv.com/dwm93.
