# Supplementary material for: Effect of sweet and caloric drinks on cardiac reactivity to slow-paced breathing in healthy adults
Source: Sci Rep. 2025 May 19;15:17368. doi: 10.1038/s41598-025-00980-w (PMC12089329; doi:10.1038/s41598-025-00980-w)
Supplement: Supplementary file 1 — Supplementary Material 1 [file 41598_2025_980_MOESM1_ESM.docx]

*Supplements for* Effect of sweet and caloric drinks on cardiac reactivity to slow paced breathing in healthy adults

Maria Meier^1,2^, Stephanie J. Dimitroff^3^, Bernadette F. Denk^1,4^, Eva Unternaehrer^2^ & Jens C. Pruessner^1,4^

1. University of Konstanz, Konstanz, Germany
2. Child- and Adolescent Psychiatric Research Department, University Psychiatric Clinics Basel (UPK), University of Basel, Basel Switzerland
3. University of Montana, Montana, United States
4. Centre for the Advanced Study of Collective Behavior, Konstanz, Germany

* Correspondence: Maria Meier ([maria.meier@uni-konstanz.de](mailto:maria.meier@uni-konstanz.de))

*Table S1.* Comparison of nested models predicting blood glucose concentration.

|  | *model* | *df* | *AIC* | *BIC* | *Log likelihood* | *Test* | *Log likelihood ratio* | *p-value* |
| --- | --- | --- | --- | --- | --- | --- | --- | --- |
| Intercept | 1 | 2 | 4696.06 | 4704.33 | -2346.03 |  |  |  |
| Random intercept | 2 | 3 | 4600.41 | 4612.81 | -2297.21 | 1 vs 2 | 97.6503 | <.001 |
| Random slope | 3 | 5 | 4157.02 | 4177.68 | -2073.51 | 2 vs 3 | 447.391 | <.001 |
| Time | 4 | 6 | 4099.16 | 4123.94 | -2043.58 | 3 vs 4 | 59.8666 | <.001 |
| Time^2^ | 5 | 7 | 4081.64 | 4110.56 | -2033.82 | 4 vs 5 | 19.5202 | <.001 |
| condition | 6 | 10 | 4053.02 | 4094.34 | -2016.51 | 5 vs 6 | 34.6126 | <.001 |
| Condition x time | 7 | 16 | 3845.14 | 3911.24 | -1906.57 | 6 vs 7 | 219.888 | <.001 |

*Table S2.* Evaluation of the mixed model predicting blood glucose concentrations.

|  | **value** | | | |  |
| --- | --- | --- | --- | --- | --- |
| *Predictors* | *Estimates* | *CI* | | *p* | |
| (Intercept) | 92.41 | 87.30 – 97.53 | | **<0.001** | |
| time [1st degree] | -29.71 | -93.57 – 34.14 | | 0.367 | |
| time [2nd degree] | 16.57 | -25.65 – 58.80 | | 0.447 | |
| drink content [sweet] | 14.07 | 6.44 – 21.70 | | **<0.001** | |
| drink content [calories] | 59.38 | 51.67 – 67.09 | | **<0.001** | |
| drink content [sweet & calories] | 56.89 | 49.72 – 64.07 | | **<0.001** | |
| time [1st degree] × drink content [sweet] | -2.30 | -96.85 – 92.24 | | 0.962 | |
| time [2nd degree] × drink content [sweet] | -36.23 | -98.75 – 26.29 | | 0.261 | |
| time [1st degree] × drink content [calories] | 713.84 | 618.27 – 809.40 | | **<0.001** | |
| time [2nd degree] × drink content [calories] | -60.45 | -123.65 – 2.75 | | 0.064 | |
| time [1st degree] × drink content [sweet & calories] | 728.33 | 639.41 – 817.25 | | **<0.001** | |
| time [2nd degree] × drink content [sweet & calories] | -172.55 | -231.35 – -113.74 | | **<0.001** | |
| **Random Effects** | | |  |  |  |
| σ^2^ | 124.23 | | | |  |
| τ_00_ _code_ | 105.72 | | | |  |
| τ_11_ _code.time_ | 0.24 | | | |  |
| ρ_01_ _code_ | -0.06 | | | |  |
| ICC | 0.46 | | | |  |
| N _code_ | 115 | | | |  |
| Observations | 460 | | | |  |
| Marginal R^2^ / Conditional R^2^ | 0.843 / 0.915 | | | |  |

*Table S3.* Comparison of nested models predicting RMSSD.

|  | *model* | *df* | *AIC* | *BIC* | *Log likelihood* | *Test* | *Log likelihood ratio* | *p-value* |
| --- | --- | --- | --- | --- | --- | --- | --- | --- |
| intercept | 1 | 2 | 5386.615 | 5395.324 | -2691.308 |  |  |  |
| Random intercept | 2 | 3 | 5078.372 | 5091.435 | -2536.186 | 1 vs 2 | 310.24356 | <.001 |
| Time | 3 | 6 | 5065.393 | 5091.519 | -2526.696 | 2 vs 3 | 18.97884 | .0003 |
| Time^2^ | 4 | 7 | 5051.618 | 5082.098 | -2518.809 | 3 vs 4 | 15.77528 | .0001 |
| Time^2^ | 5 | 8 | 5047.817 | 5082.652 | -2515.909 | 4 vs 5 | 5.80013 | .0160 |
| Auto regressive covariance structure (AR1) | 6 | 9 | 5041.381 | 5080.571 | -2511.691 | 5 vs 6 | 8.43594 | .0037 |
| covariates | 7 | 11 | 5040.254 | 5088.153 | -2509.127 | 6 vs 7 | 5.12699 | .0770 |
| condition | 8 | 14 | 5037.948 | 5098.909 | -2504.974 | 7 vs 8 | 8.30658 | .0401 |
| Condition x time | 9 | 23 | 5043.200 | 5143.351 | -2498.600 | 8 vs 9 | 12.74755 | .1744 |

*Table S4.* Evaluation of the mixed model predicting RMSSD.

|  | **value** | |  |
| --- | --- | --- | --- |
| *Predictors* | *Estimates* | *CI* | *p* |
| (Intercept) | 81.99 | 40.61 – 123.37 | **<0.001** |
| time [1st degree] | 73.47 | 19.38 – 127.57 | **0.009** |
| time [2nd degree] | -84.27 | -137.92 – -30.62 | **0.003** |
| time [3rd degree] | 5.98 | -51.91 – 63.87 | 0.842 |
| humidity | -0.31 | -0.69 – 0.06 | 0.106 |
| temperature | -0.55 | -2.52 – 1.41 | 0.584 |
| drink content [sweet] | -7.66 | -19.02 – 3.69 | 0.191 |
| drink content [calories] | -7.50 | -19.80 – 4.80 | 0.237 |
| drink content [sweet & calories] | -14.99 | -25.21 – -4.78 | **0.005** |
| time [1st degree] × drink content [sweet] | -41.86 | -121.97 – 38.24 | 0.313 |
| time [2nd degree] × drink content [sweet] | 21.07 | -58.36 – 100.51 | 0.608 |
| time [3rd degree] × drink content [sweet] | -42.43 | -128.14 – 43.28 | 0.339 |
| time [1st degree] × drink content [calories] | -34.36 | -115.33 – 46.60 | 0.412 |
| time [2nd degree] × drink content [calories] | 68.93 | -11.36 – 149.23 | 0.097 |
| time [3rd degree] × drink content [calories] | -22.26 | -108.90 –  64.39 | 0.620 |
| time [1st degree] × drink content [sweet & calories] | 10.25 | -65.09 – 85.59 | 0.792 |
| time [2nd degree] × drink content [sweet & calories] | 58.07 | -16.64 – 132.78 | 0.133 |
| time [3rd degree] × drink content [sweet & calories] | -98.44 | -179.06 – -17.82 | **0.019** |
| **Random Effects** | | |  |
| σ^2^ | 209.53 | |  |
| τ_00_ _nummer_ | 556.80 | |  |
| τ_11_ _nummer.time_ | 2.67 | |  |
| ρ_01_ _nummer_ | -0.84 | |  |
| ICC | 0.73 | |  |
| N _nummer_ | 115 | |  |
| Observations | 575 | |  |
| Marginal R^2^ / Conditional R^2^ | 0.086 / 0.750 | |  |

*S5.* Comparison of nested models predicting PEP.

|  | *model* | *df* | *AIC* | *BIC* | *Log likelihood* | *Test* | *Log likelihood ratio* | *p-value* |
| --- | --- | --- | --- | --- | --- | --- | --- | --- |
| intercept | 1 | 2 | 4240.5 | 4249.06 | -2118.25 |  |  |  |
| Random intercept | 2 | 3 | 3881 | 3893.85 | -1937.5 | 1 vs 2 | 361.492 | <.0001 |
| Time | 3 | 4 | 3878.95 | 3896.08 | -1935.48 | 2 vs 3 | 4.0518 | 0.0441 |
| Time^2^ | 4 | 5 | 3787.39 | 3808.8 | -1888.69 | 3 vs 4 | 93.5647 | <.0001 |
| Time^2^ | 5 | 6 | 3783.69 | 3809.38 | -1885.84 | 4 vs 5 | 5.6994 | 0.017 |
| covariates | 6 | 7 | 3779.7 | 3809.68 | -1882.85 | 5 vs 6 | 5.9849 | 0.0144 |
| condition | 7 | 10 | 3783.37 | 3826.19 | -1881.68 | 6 vs 7 | 2.336 | 0.5057 |
| Condition x time | 8 | 16 | 3768.78 | 3837.29 | -1868.39 | 7 vs 8 | 26.5897 | 0.0002 |

*Table S6.* Evaluation of the mixed model predicting PEP.

|  | **value** | | |  |
| --- | --- | --- | --- | --- |
| *Predictors* | *Estimates* | *CI* | *p* |  |
| (Intercept) | 101.14 | 92.78 – 109.51 | **<0.001** |  |
| time [1st degree] | 17.36 | -6.33 – 41.05 | 0.156 |  |
| time [2nd degree] | 70.67 | 47.83 – 93.52 | **<0.001** |  |
| humidity | 0.14 | -0.04 – 0.33 | 0.136 |  |
| drink content [sweet] | -2.33 | -8.39 – 3.73 | 0.453 |  |
| drink content [calories] | -5.10 | -11.72 – 1.52 | 0.134 |  |
| drink content [sweet & calories] | -2.14 | -7.44 – 3.16 | 0.431 |  |
| time [1st degree] × drink content [sweet] | 2.03 | -34.40 – 38.45 | 0.914 |  |
| time [2nd degree] × drink content [sweet] | -22.51 | -57.63 – 12.61 | 0.214 |  |
| time [1st degree] × drink content [calories] | -62.80 | -99.23 – -26.38 | **0.001** |  |
| time [2nd degree] × drink content [calories] | 8.31 | -26.81 – 43.43 | 0.646 |  |
| time [1st degree] × drink content [sweet & calories] | -62.57 | -95.30 – -29.83 | **<0.001** |  |
| time [2nd degree] × drink content [sweet & calories] | -15.85 | -47.41 – 15.72 | 0.330 |  |
| **Random Effects** | | | | |
| σ^2^ | 38.23 | | |  |
| τ_00_ _nummer_ | 103.33 | | |  |
| N _nummer_ | 107 | | |  |
| Observations | 535 | | |  |
| Marginal R^2^ / Conditional R^2^ | 0.333 / NA | | |  |

*Table S7.* Comparison of nested models predicting subjective relaxation.

|  | *model* | *df* | *AIC* | *BIC* | *Log likelihood* | *Test* | *Log likelihood ratio* | *p-value* |
| --- | --- | --- | --- | --- | --- | --- | --- | --- |
| intercept | 1 | 2 | 5953.78 | 5962.85 | -2974.89 |  |  |  |
| Random intercept | 2 | 3 | 5902.54 | 5916.15 | -2948.27 | 1 vs 2 | 53.2392 | <.0001 |
| Time | 3 | 4 | 5900.81 | 5918.95 | -2946.4 | 2 vs 3 | 3.73083 | 0.0534 |
| Time^2^ | 4 | 5 | 5855.4 | 5878.09 | -2922.7 | 3 vs 4 | 47.4008 | <.0001 |
| Time^2^ | 5 | 6 | 5805.06 | 5832.28 | -2896.53 | 4 vs 5 | 52.3402 | <.0001 |
| condition | 6 | 9 | 5807.29 | 5848.12 | -2894.64 | 5 vs 6 | 3.77716 | 0.2866 |
| Condition x time | 7 | 18 | 5818.97 | 5900.63 | -2891.48 | 6 vs 7 | 6.32145 | 0.7074 |

*Table S8.* Evaluation of the mixed model predicting subjective relaxation.

|  | **value** | | |
| --- | --- | --- | --- |
| *Predictors* | *Estimates* | *CI* | *p* |
| (Intercept) | 42.65 | 38.98 – 46.31 | **<0.001** |
| time [1st degree] | 6.37 | -48.74 – 61.48 | 0.823 |
| time [2nd degree] | 106.55 | 51.44 – 161.66 | **<0.001** |
| time [3rd degree] | 110.33 | 55.22 – 165.44 | **<0.001** |
| drink content [sweet] | -5.41 | -10.88 – 0.06 | 0.055 |
| drink content [calories] | -2.54 | -8.07 – 2.99 | 0.370 |
| drink content [sweet & calories] | -2.51 | -7.65 – 2.64 | 0.342 |
| time [1st degree] × drink content [sweet] | 56.98 | -24.62 – 138.58 | 0.176 |
| time [2nd degree] × drink content [sweet] | 34.89 | -46.71 – 116.49 | 0.407 |
| time [3rd degree] × drink content [sweet] | 28.47 | -53.13 – 110.07 | 0.498 |
| time [1st degree] × drink content [calories] | 22.81 | -59.68 – 105.29 | 0.592 |
| time [2nd degree] × drink content [calories] | -38.08 | -120.56 – 44.40 | 0.371 |
| time [3rd degree] × drink content [calories] | -25.72 | -108.20 – 56.77 | 0.545 |
| time [1st degree] × drink content [sweet & calories] | 23.35 | -53.40 – 100.09 | 0.555 |
| time [2nd degree] × drink content [sweet & calories] | 5.56 | -71.19 – 82.31 | 0.888 |
| time [3rd degree] × drink content [sweet & calories] | -9.57 | -86.32 – 67.18 | 0.809 |
| **Random Effects** | | |  |
| σ^2^ | 212.22 | | |
| τ_00_ _nummer_ | 72.36 | | |
| N _nummer_ | 115 | | |
| Observations | 690 | | |
| Marginal R^2^ / Conditional R^2^ | 0.161 / NA | | |
